# Incidence and associated factors of extrauterine growth restriction (EUGR) in preterm infants, a cross-sectional study in selected NICUs in Ethiopia

**DOI:** 10.1136/bmjpo-2020-000765

**Published:** 2020-10-12

**Authors:** Netsanet Workneh Gidi, Robert L Goldenberg, Assaye K Nigussie, Elizabeth McClure, Amha Mekasha, Bogale Worku, Matthias Siebeck, Orsolya Genzel-Boroviczeny, Lulu M Muhe

**Affiliations:** 1 CIHLMU Center for International Health, Ludwig‐Maximilians‐Universität, Munich, Germany; 2 Department of Pediatrics and Child Health, Jimma University, Jimma, Ethiopia; 3 Department of Obstetrics and Gynecology, Columbia University, New York, New York, USA; 4 Newborn & Child Health, Bill and Melinda Gates Foundation, Seattle, Washington, USA; 5 Center for Clinical Research Network Coordination, RTI International, Research Triangle Park, North Carolina, USA; 6 Department of Pediatrics and Child Health, Addis Ababa University College of Health Sciences, Addis Ababa, Oromia, Ethiopia; 7 Ethiopian Pediatric Society, Addis Ababa, Ethiopia; 8 Institute for Medical Education, University Hospital, LMU Munich, Germany, Munich, Germany; 9 Dr. von Hauner University Children’s Hospital, Ludwig Maximilian University of Munich, München, Germany

**Keywords:** neonatology

## Abstract

**Background:**

Preterm infants have high risk of developing growth restriction and long-term complications. Enteral feeding is often delayed in neonatal intensive care units (NICUs) for the fear of feeding intolerance and the associated necrotising enterocolitis, and recent advances in nutritional support are unavailable in low-income countries.

**Objective:**

The aim of this study was to assess the incidence and associated factors of extrauterine growth restriction (EUGR) among preterm infants in selected NICUs in Ethiopia.

**Method:**

This was a cross-sectional study involving a subgroup analysis of preterm infants admitted to hospitals, from a multicentre descriptive study of cause of illness and death in preterm infants in Ethiopia, conducted from 2016 to 2018. EUGR was defined as weight at discharge Z-scores <−1.29 for corrected age. Clinical profiles of the infants were analysed for associated factors. SPSS V.23 software was used for analysis with a significance level of 5% and 95% CI.

**Result:**

From 436 preterm infants included in the analysis, 223 (51%) were male, 224 (51.4%) very low birth weight (VLBW) and 185 (42.4%) small for gestational age (SGA). The mean (SD) of weight for corrected age Z-score at the time of discharge was −2.5 (1.1). The incidence of EUGR was 86.2%. Infants who were SGA, VLBW and longer hospital stay over 21 days had increased risk of growth restriction (p-value<0.01). SGA infants had a 15-fold higher risk of developing EUGR at the time of discharge from hospital than those who were appropriate or large for gestational age (OR (95% CI)=15.2 (4.6 to 50.1).

**Conclusion:**

The majority of the infants had EUGR at the time of discharge from the hospital, which indicates suboptimal nutrition. Revision of national guidelines for preterm infants feeding and improvement in clinical practice is highly required.

**What is known about the subject?**

- Preterm infants’ growth is expected to be similar to that of the intrauterine foetus, however small preterm infants often develop extrauterine growth restriction (EUGR).
- EUGR is associated with increased risk of post-neonatal mortality and long-term morbidities such as adverse metabolic and neurodevelopmental outcomes in subsequent years.
- There is paucity of data regarding preterm nutrition in low-income and middle-income countries; parenteral nutrition and use of human milk fortifiers are often unavailable.

**What this study adds?**

- Neonatal mortality rate of hospital admitted preterm infants in Ethiopia is 29%, 86.2% of the infants who survived the immediate complications were discharged with extrauterine growth restriction (EUGR).
- The high incidence of EUGR indicates insufficient nutritional support of the preterm infants.
- Infants with very low birth weight, hospital stay over 21 days and small for gestational age had increased risk of developing EUGR.

## Introduction

Complications of preterm births are the leading causes of newborn deaths worldwide. Survivors of preterm birth are at increased risk of adverse metabolic and neurodevelopmental long-term outcomes.^1^ Ideally, growth of the preterm infant is expected to be similar to that of the intrauterine foetus at the same gestational age (GA) once birth weight has been regained. However, attaining that goal requires optimal nutritional support to address the increased needs of nutrients for catch-up growth.^2 3^ Extrauterine growth restriction (EUGR) is a severe nutritional deficit during the first weeks after birth, commonly seen in small preterm infants.^4 5^ Factors associated with EUGR reported from developed countries include caloric and protein deficits, intrauterine growth restriction (IUGR), neonatal morbidities and the need for prolonged hospital stay.^6^ EUGR is commonly defined as a growth measurement that is <10th percentile of the predicted value at the time of hospital discharge.^5^

Improved nutritional support may decrease the rate of EUGR. This support may include early administration of parenteral nutrition, use of human milk fortifiers and preterm formula when mother’s milk is unavailable.^7 8^ Identifying infants at risk of growth failure by monitoring weight and nutritional intake should guide clinicians to increase nutritional support that is individualised according to the need of the preterm infant.^9^ Many mothers of preterm infants need support to produce and express enough milk as the babies are often too weak to suckle.^10 11^ Recent evidence indicates that early, fast or continuous enteral feeding results in better neonatal outcomes compared with late, slow or intermittent feeding.^10 12^ However, clinicians in many NICUs often delay enteral feeding for the fear of feeding intolerance and the associated necrotising enterocolitis (NEC).^13^

For LMICs WHO guidelines on feeding stable low birth weight infants whose birth weight is greater than 1000 g recommend feeding mother’s own milk starting from the first day. Those who cannot be fed mother’s own milk should be fed donor human milk (when available); if this is not possible standard infant formula has to be given, those who fail to gain weight despite adequate feeding with standard infant formula should be given preterm infant formula. And very low birth weight (VLBW) infants who fail to gain weight despite adequate breast milk feeding should be given human-milk fortifiers.^14^ In Ethiopian neonatal guideline, mothers breast milk is the only option recommended for feeding preterm infants; however, the use of donors milk, standard infant formula milk, preterm formula milk and human milk fortifiers were not considered as options where indicated.^15^ EUGR is associated with long-term morbidities such as adverse neurodevelopmental outcomes in subsequent years. Hence, assessment of the magnitude of the problem and recognising associated factors should help to identify and manage preterm infants at risk of growth restriction and consequently improve long-term outcomes.^16^ The burden of preterm birth is increasing worldwide, and the highest average rate of preterm birth occurs in low-income countries (11.8%).^17^ However, there is paucity of data regarding preterm nutrition and EUGR in low-income and middle-income countries, most of the literatures in this area are reported from high-income countries. Thus, the aim of this study is to assess the incidence and associated factors of EUGR in preterm infants in five NICUs in Ethiopia.

## Methods

### Data source

This is a cross-sectional study involving the analysis of a subgroup of 436 preterm infants from a multicentre descriptive study conducted in five selected hospitals, ‘Study of causes of illness and death in preterm infants (SIP)’. The primary study and methodology papers have been previously published.^18 19^ The hospital practice of neonatal care was based on a national neonatal guideline. Stable preterm infants are fed on mothers own breast milk. For infants weighing <1.5 kg at birth, starting expressed breast milk 10 mL/kg per day and increasing the amount by 20 mL/kg/day according to the infants’ condition until full volume feeding is achieved. The goal is to achieve, volume: 140–150 mL/kg/day and calorie: 110–120 kcal/kg/day. Other nutritional support methods, such as the use of donor milk, parenteral nutrition and breast milk fortification were not available.^15^

Preterm infants with a GA of 28–36 weeks, who were discharged alive from the hospitals, were considered for the analysis. The exclusion criteria included infants with a congenital malformation, chromosomal abnormalities; those who died before discharge from the hospital, and had a hospital stay less of than 2 weeks. GA estimation was done by a combination of last menstrual period, ultrasound and New Ballard Score assessment. Variables such as birth weight, discharge weight, estimated GA, clinical profile of the infants, corrected age and duration of hospital stay were analysed.

### Statistical analysis

Weight for GA and weight for corrected age Z-scores were calculated using gender specific Fenton growth chart calculation spreadsheets.^20^ Small for gestational age (SGA) and EUGR was defined as weight for GA and weight at discharge for corrected age <−1.29 or less than the 10th percentile, respectively.

Statistical analyses were done using SPSS V.23 software. Following descriptive analysis, the χ^2^ test was used to check the cell count adequacy before performing univariate logistic regression. Factors that could be associated with the dependent variables were identified from univariate logistic regression (p-value<0.2). Stepwise multivariate logistic regression was performed to identify independent risk factors for EUGR with significance level of 5% and 95% CI.

### Patient and public involvement statement

Study participants were not involved in the design of the study.

## Results

**Figure 1:**
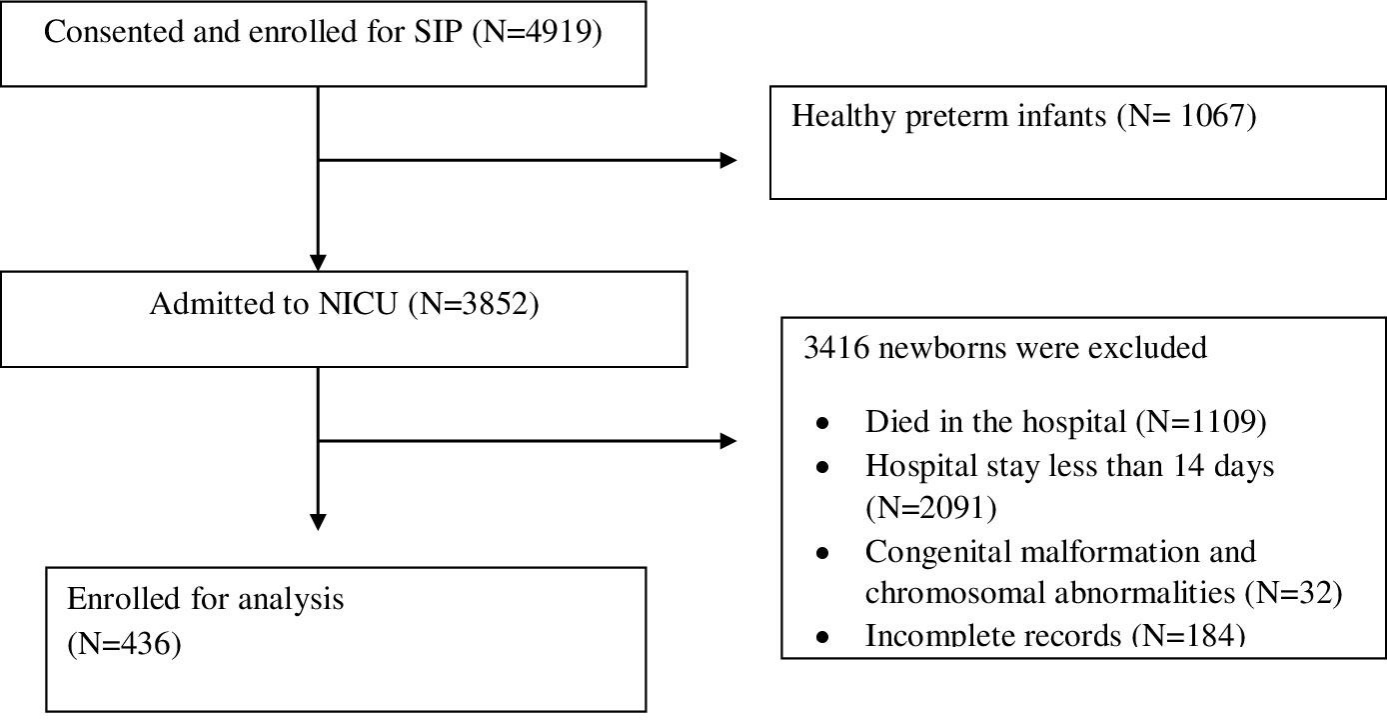
Flow chart of study subjects included in the analysis. NICU neonatal intensive care units; SIP study of illness in preterms.

Figure 1 shows the flow chart of recruitment of study subjects, including those who were excluded because they were discharged early, died in the hospital or had chromosomal abnormalities and congenital malformations. A total of 436 preterm infants were eligible for the analysis, 223 (51%) were male. Nearly half 205 (47%) of the infants were very preterm (born at GA of 28–32 weeks) and 224 (51.4%) were VLBW (birth weight less than 1500 g). The rate of small for GA (SGA) among the study subjects was 42.4%, while 55.5% and 2.1% were appropriate for GA (AGA) and large for GA (LGA), respectively. The birth weight for GA Z-score, mean (SD) was −1.1 (1.0), while weight for corrected age Z-score mean (SD) at the time of discharge was −2.5 (1.1) (table 1).

**Table 1:** Clinical characteristics of the preterm infants

Variables	Values
Female/male ratio (**%**)	49/51
GA (weeks), no. (%)	
28–32	205 (47.0)
32–34	148 (33.9)
35–<37	83 (19.0)
Birth weight (g), no. (%)	
<1000	28 (6.4)
1000–1500	196 (45.0)
1500–2000	164 (37.6)
>2000	48 (11.0)
Weight for gestational age, no. (%)	
AGA	242 (55.5)
LGA	9 (2.1)
SGA	185 (42.4)
Pregnancy	
Singleton	260 (59.6)
Twins	166 (38.1)
Triplets	10 (2.3)
Weight for gestational age Z-score at birth, mean (SD)	−1.1 (1.0)
Weight for corrected age Z-score at discharge, mean (SD)	−2.5 (1.1)
Newborn major diagnosis, no. (%)*	
Neonatal infections	214 (49.1)
Respiratory distress syndrome	190 (43.6)
Feeding problems	111 (25.6)
Perinatal asphyxia	19 (4.4)
Hypothermia	253 (58.0)
Anaemia	82 (18.8)
Total duration of hospital stay, mean days (SD)	21.5 (5.1)
Corrected age at discharge, week, mean (SD)	35.4 (1.9)

*The percent does not add up to 100 since the infants may have had more than one diagnosis.

AGA, appropriate for gestational age; GA, gestational age; LGA, large for gestational age; SGA, small for gestational age.

Nearly half of the infants, 214 (49.1%) had neonatal infections such as neonatal sepsis, pneumonia, meningitis and NEC, while 190 (43.6%), 111 (25.6%), 253 (58%) and 19 (4.4%) had respiratory distress syndrome, feeding problems, hypothermia and perinatal asphyxia, respectively. The mean (SD) duration of hospital stay was 21.5 (5.1) days and the mean (SD) of corrected age at discharge was 35.4 (1.9) weeks.

The overall incidence of EUGR was 86.2%. Almost all (98.4%) of the infants born SGA had EUGR at discharge, while fewer of the LGA cases (22.2%) were classified as EUGR at the time of discharge from the hospital. Comparable rates of EUGR were observed across the infants’ major diagnoses. Birth weight, weight for GA and duration of hospital stay were found to be associated with the occurrence of EUGR (p-value<0.01) (table 2). Variables associated with a statistically significant increased risk of EUGR on univariate logistic regression include being SGA, VLBW and duration of hospital stay over 21 days. Similarly, on stepwise multivariate logistic regression, SGA, VLBW and longer hospital stay over 21 days were found to be independent risk factors for EUGR. SGA infants had a 15-fold increased risk of developing EUGR at the time discharge from hospital than those who were AGA or LGA (OR (95% CI)=15.2 (4.6 to 50.1) (table 3).

**Table 2:** Univariate logistic regression, factors associated with extrauterine growth restriction (EUGR)

Variables	Total no.	EUGR cases no. (%)	P value	OR (95% CI)
Overall incidence of EUGR	436	376 (86.2)	–	–
Gender				
Female	213	191 (86.9)	–	–
Male	223	185 (85.6)	0.71	0.9 (0.5 to 1.6)
Birth weight				
<1500	224	210 (93.8)	0<0.001	4.9 (2.2 to 11.1)
>1500	212	166 (78.3)	–	–
Weight for GA				
AGA and LGA	251	194 (77.3)	–	–
SGA	185	182 (98.4)	0<0.001	17.8 (5.4 to 57.9)
Pregnancy				
Singleton	260	226	–	–
Twins and triplets	176	150	0.61	1.2 (0.7 to 2.0)
Major diagnosis of the preterm infants				
Infection	214	184 (86.0)	0.87	1.0 (0.55 to 1.65)
Respiratory distress syndrome	190	162 (85.3)	0.60	0.8 (0.5 to 1.5)
Perinatal asphyxia	19	16 (84.2)	0.79	0.8 (0.2 to 2.9)
Feeding problems	131	111 (84.7)	0.55	0.8 (0.6 to 1.5)
Anaemia	82	67 (81.7)	0.18	0.7 (0.3 to 1.2)
Duration of hospital stay				
14–21 days	224	179 (79.9)	–	–
>21 days	212	197 (92.9)	0<0.001	3.3 (1.8 to 6.1)

AGA, appropriate for gestational age; EUGR, extrauterine growth restriction; GA, gestational age; LGA, large for gestational age; SGA, small for gestational age.

**Table 3:** Multivariate logistic regression analysis, independent risk factors of extrauterine growth restriction

Variables	P value	AOR (95% CI)
Birth weight		
Non-SGA	–	–
SGA	<0.001	15.2 (4.6 to 50.1)
Weight for GA		
≥1500 g	–	–
<1500 g	0.03	2.2 (1.1 to 4.3)
Duration of hospital stay		
<21 days	–	–
≥21 days	<0.001	2.7 (1.4 to 5.3)

AOR, adjusted OR; EUGR, extrauterine growth restriction; GA, gestational age; SGA, small for gestational age.

## Discussion

The SIP study has showed a very high mortality rate (29%) among hospital admitted preterm infants.^19^ The current follow-up study revealed that most (86.2%) of the infants who survived the immediate complications were discharged with EUGR and associated severe caloric and protein deficits. Establishing adequate dietary intakes in preterm infants is a very common problem in NICUs; however, optimal nutrition is critically important to insure survival, normal growth and development in subsequent years.^21 22^

The incidence of EUGR observed in this study was comparable to the 89% EUGR rate in extremely low birth weight infants (birth weight less than 1000 gm) reported by Dusick *et al* from the USA,^23^ however only 6.4% of the study population in the current study were extremely low birth weight. The rate of EUGR in this study was much higher than that reported from China by Shan *et al*; Lima *et al* from Brazil and Clark *et al* from the USA, 56.8%,^24^ 26%,^6^ 28%,^5^ respectively. Nearly half the preterm infants in the current study had a birth weight greater than 1500 g, while the other studies included mainly VLBW infants and those with extreme prematurity. In addition, our study did not include infants with a GA less than 28 weeks. Thus, our study shows a rate of EUGR that was unacceptably high in higher GA preterm infants.

The mean Z-score of birth weight and weight at discharge in this study was significantly lower than the averages reported in other literature.^6 25^ Shan *et al* have shown risk factors related to EUGR, such as male gender, low GA at birth, low birth weight and long length of hospital stay.^24^ In this study, we found increased risk of EUGR in infants who were SGA, VLBW and hospitalised over 21 days (table 3). Sakurai *et al* from Japan have also reported lower GA, IUGR, severe chronic lung disease and poor nutrition as relevant risk factors associated with EUGR, the SGA infants in our study are likely to have had IUGR, but none of the comorbidities the infants had was associated with increased risk EUGR.^26^ Generally, preterm infants born SGA have higher risk of morbidity and mortality compared with AGA preterm infants.^6 27^ The rate of EUGR was higher (92.9%) in infants who stayed in the hospital over 21 days. This is probably due to the severity of the infants’ morbidities and the inadequacy of nutritional support provided in the NICUs.

Aggressive nutritional support has been shown to promote growth without increased risk of adverse effects.^28^ With optimal nutrition, postnatal growth failure could be prevented and extrauterine weight gain can be achieved similar to fetuses of same GA.^29^ A combination of parenteral nutrition, early advancement of enteral feeding and fortification of human milk are the current standards of care in developed countries.^3 30^ These interventions are often not available in low-income countries. Under-nutrition experienced in infancy is known to impair cognitive function, school achievement and results in increased risk of behavioural problems later in life.^31^

This study has several limitations, the mean corrected age at discharge was 35.4 weeks, and follow-up at around 40 weeks could have possibly shown catch up growth. We used similar definition of EUGR for all infants in the study, SGA infants’ growth velocity was not considered for diagnosis EUGR. Nutritional data, maternal conditions and delivery relating factors were not assessed as risk factors. This was a cross-sectional study design, the main aim was to assess the incidence of EUGR, associated factors were analysed with the available data. Further study is required to identify all the predictors of EUGR.

## Conclusion

The high incidence of EUGR observed in this study indicates that the nutritional support of the preterm infants was insufficient. The risk of developing EUGR was higher in preterm infants who were VLBW, SGA and hospitalised for over 21 days. Much attention needs to be given to improve preterm nutrition in low-income countries. Country infant feeding guidelines need revision based on recent evidences to improve preterm nutrition. Regular monitoring of nutritional status and individualised timely nutritional intervention has to be the standard of care of preterm infants in the NICUs.

## Supplementary Material

Author's manuscript
