# Supplementary material for: Incidence and associated factors of extrauterine growth restriction (EUGR) in preterm infants, a cross-sectional study in selected NICUs in Ethiopia
Source: BMJ Paediatr Open. 2020 Oct 12;4(1):e000765. doi: 10.1136/bmjpo-2020-000765 (PMC7552851; doi:10.1136/bmjpo-2020-000765)
Supplement: Author's manuscript [file bmjpo-2020-000765.draft_revisions.pdf]

BMJ Paediatrics Open

BMJ Paediatrics Open is committed to open peer review. As part of this commitment we make the peer review history of every article we publish publicly available.

When an article is published we post the peer reviewers' comments and the authors' responses online. We also post the versions of the paper that were used during peer review. These are the versions that the peer review comments apply to.

The versions of the paper that follow are the versions that were submitted during the peer review process. They are not the versions of record or the final published versions. They should not be cited or distributed as the published version of this manuscript.

BMJ Paediatrics Open is an open access journal and the full, final, typeset and author-corrected version of record of the manuscript is available on our site with no access controls, subscription charges or pay-per-view fees (http://bmjpaedsopen.bmj.com).

If you have any questions on BMJ Paediatrics Open's open peer review process please email info.bmjpo@bmj.com

BMJ Paediatrics Open

Incidence and associated factors of extrauterine growth restriction (EUGR) in preterm infants, a cross-sectional study in selected NICUs in Ethiopia

Journal:	*BMJ Paediatrics Open*
Manuscript ID	bmjpo-2020-000765
Article Type:	Original research
Date Submitted by the Author:	14-Jun-2020
Complete List of Authors:	Gidi, Netsanet; Jimma University College of Public Health and Medical Sciences, Pediatric and child health<br>Goldenberg , Robert L. ; Columbia University<br>Nigussie, Assaye K. ; Bill and Melinda Gates Foundation<br>McClure, Elizabeth; RTI International<br>Mekasha, Amha; Addis Ababa University College of Health Sciences<br>Worku, Bogale; Ethiopian Pediatric Society<br>Siebeck, Matthias; University Hospital, LMU Munich, Germany, Institute for Medical Education<br>Genzel-Boroviczeny, Orsolya; Ludwig Maximilian University of Munich , Dr. von Hauner University Children's Hospital<br>Muhe, Lulu M; Addis Ababa University College of Health Sciences
Keywords:	Neonatology

SCHOLARONE™
Manuscripts

I, the Submitting Author has the right to grant and does grant on behalf of all authors of the Work (as defined in the below author licence), an exclusive licence and/or a non-exclusive licence for contributions from authors who are: i) UK Crown employees; ii) where BMJ has agreed a CC-BY licence shall apply, and/or iii) in accordance with the terms applicable for US Federal Government officers or employees acting as part of their official duties; on a worldwide, perpetual, irrevocable, royalty-free basis to BMJ Publishing Group Ltd ("BMJ") its licensees and where the relevant Journal is co-owned by BMJ to the co-owners of the Journal, to publish the Work in this journal and any other BMJ products and to exploit all rights, as set out in our licence.

The Submitting Author accepts and understands that any supply made under these terms is made by BMJ to the Submitting Author unless you are acting as an employee on behalf of your employer or a postgraduate student of an affiliated institution which is paying any applicable article publishing charge ("APC") for Open Access articles. Where the Submitting Author wishes to make the Work available on an Open Access basis (and intends to pay the relevant APC), the terms of reuse of such Open Access shall be governed by a Creative Commons licence – details of these licences and which Creative Commons licence will apply to this Work are set out in our licence referred to above.

Other than as permitted in any relevant BMJ Author's Self Archiving Policies, I confirm this Work has not been accepted for publication elsewhere, is not being considered for publication elsewhere and does not duplicate material already published. I confirm all authors consent to publication of this Work and authorise the granting of this licence.

Confidential: For Review Only

Title: Incidence and associated factors of extrauterine growth restriction (EUGR) in preterm infants, a cross-sectional study in selected NICUs in Ethiopia

Authors: Dr. Netsanet Workneh Gidi[a,i], Dr. Robert L. Goldenberg[b], Prof. Assaye K. Nigussie[c], Dr. Elizabeth M. McClure[d], Prof. Amha Mekasha[e], Prof. Bogale Worku[f], Prof. Dr. Matthias Siebeck[g], Prof. Dr. Orsolya Genzel-Boroviczény[h], Prof. Lulu M. Muhe[e]

Affiliations

[a] Institute of Health Sciences, Jimma University, Jimma, Ethiopia

[b] Columbia University, New York, NY, USA

[c] Bill and Melinda Gates Foundation, Seattle, WA, USA

[d] RTI International, 3040 Cornwallis Rd., Durham, NC, 27709, USA

[e] College of Health Sciences, Addis Ababa University, Addis Ababa, Ethiopia

[f] Ethiopian Pediatric Society, Addis Ababa, Ethiopia

[g] Institute for Medical Education, University Hospital, LMU Munich, Germany

[h] Dr von Hauner Childrens´Hospital, University Hospital, LMU Munich, Germany

[i] Center for International Health, University Hospital, LMU Munich, Germany

https://mc.manuscriptcentral.com/bmjpo

Corresponding author: Netsanet Workneh Gidi (MD, DTM&H)

Associate professor of pediatrics and child health

College of public health and medical sciences, Jimma University, Jimma, Ethiopia

PhD candidate at CIHLMU, Munich, Germany

Phone: +251 917762109

Email: konetsanet@gmail.com, or Netsanet.Workneh@lrz.uni-muenchen.de

Confidential: For Review Only

Abstract

Background: Preterm infants have high risk of developing growth restriction and long term complications. Establishing enteral feeding is often difficult in neonatal intensive care units, and recent advances in nutritional support are unavailable in low income countries.

Objective: The aim of this study was to assess the incidence and associated factors of extrauterine growth restriction (EUGR) among preterm infants in selected NICUs in Ethiopia.

Method: This was a cross-sectional study involving a sub-group analysis of preterm infants admitted to hospitals, from a multicenter descriptive study of cause of illness and death in preterm infants in Ethiopia. EUGR was defined as weight at discharge z-scores <-1.29 for corrected age. Clinical profiles of the infants were analyzed for associated factors. SPSS version 23 software was used for analysis with a significance level of 5% and 95% confidence interval.

Result: From 436 preterm infants included in the analysis, 223(51%) were male, 224 (51.4%) very low birth weight (VLBW) and 185(42.4%) small for gestational age (SGA). The mean (SD) of weight for corrected age Z-score at the time of discharge was -2.5 (1.1). The incidence of EUGR was 86.2%. Infants who were SGA, very low birth weight, and stayed in the hospital longer had increased risk of growth restriction (P-value <0.01). VLBW infants had a 15-fold higher risk of developing EUGR at the time discharge from hospital than those who had birth weight greater than 1500gms (odds ratio = 15.2; 95% CI, 4.6-50.1).

Conclusion: The majority of the infants had EUGR at the time of discharge from the hospital, which indicates suboptimal nutrition. Revision of national guidelines for preterm infants feeding and improvement in clinical practice is highly required.

Key words: EUGR; SGA; VLBW, preterm nutrition

What is known about the subject?

- Preterm infants' growth is expected to be similar to that of the intrauterine fetus, however small preterm infants often develop extrauterine growth restriction (EUGR).
- EUGR is associated with increased risk of post neonatal mortality and long-term morbidities such as adverse metabolic and neurodevelopmental outcomes in subsequent years.
- There is paucity of data regarding preterm nutrition in low and middle-income countries; parenteral nutrition and use of human milk fortifiers are often unavailable.

What this study adds?

Confidential: For Review Only

- Neonatal mortality rate of hospital admitted preterm infants in Ethiopia is 29%, 86.2% of the infants who survived the immediate complications were discharged with EUGR.
- The high incidence of EUGR indicates insufficient nutritional support of the preterm infants, none of the infants received human milk fortifiers or parenteral nutrition.
- Lower gestational age, very low birth weight, prolonged duration of hospital stay and small for gestational age infants had increased risk of developing EUGR.

Introduction

Complications of preterm births are the leading causes of newborn deaths worldwide. Survivors of preterm birth are at increased risk of adverse metabolic and neurodevelopmental long-term outcomes.[1] Ideally, growth of the preterm infant is expected to be similar to that of the intrauterine fetus at the same gestational age (GA) once birth weight has been regained. However, attaining that goal requires optimal nutritional support to address the increased needs of nutrients for catch-up growth.[2, 3]

Extrauterine growth restriction (EUGR) is a severe nutritional deficit during the first weeks after birth, commonly seen in small preterm infants.[4, 5] Factors associated with EUGR reported from developed countries include caloric and protein deficits, intrauterine growth restriction (IUGR), neonatal morbidities and the need for prolonged hospital stay.[6] EUGR is commonly defined as a growth measurement that is < 10th percentile of the expected intrauterine growth at the time of hospital discharge.[5]

Improved nutritional support may decrease the rate of EUGR. This support may include early administration of parenteral nutrition, use of human milk fortifiers, and preterm formula when

Confidential: For Review Only

mother's milk is unavailable.[7, 8] Identifying infants at risk of growth failure by monitoring weight and nutritional intake should guide clinicians to increase nutritional support that is individualized according to the need of the preterm infant.[9] Many mothers of preterm infants need support to produce and express enough milk as the babies are often too weak to suckle.[10,11] Recent evidence indicates that early, fast or continuous enteral feeding results in better neonatal outcomes compared to late, slow or intermittent feeding.[10, 12] However, clinicians in many neonatal intensive care units (NICUs) often delay enteral feeding for the fear of feeding intolerance and the associated necrotizing enterocolitis (NEC).[13] EUGR is associated with long-term morbidities such as adverse neurodevelopmental outcomes in subsequent years. Hence, assessment of the magnitude of the problem and recognizing associated factors should help to identify and manage preterm infants at risk of growth restriction and consequently improve long-term outcomes.[14]

The burden of preterm birth is increasing worldwide, and the highest average rate of preterm birth occurs in low-income countries (11.8%).[15] However, there is paucity of data regarding preterm nutrition and EUGR in low and middle-income countries, most of the literatures in this area are reported from high-income countries. Thus, the aim of this study is to assess the incidence and associated factors of EUGR in preterm infants in 5 NICUs in Ethiopia.

Methods

Data Source

This is a cross-sectional study involving the analysis of a sub-group of 436 preterm infants from a multicenter descriptive study conducted in five selected hospitals, "Study of causes of illness

Confidential: For Review Only

and death in preterm infants (SIP)". The primary study and methodology papers were published in Lancet Global Health and BMC Reproductive Health journals.[16, 17] The hospital practice of neonatal care was based on a national neonatal guideline.[18] Preterm infants were given breast milk, and whenever breast milk was unavailable from the mother, infant formula preparations were used. Other methods of nutritional support such as the use of donor milk, human milk fortifiers and parenteral nutrition were not practiced.

Preterm infants with a GA of 28 to 36 weeks, who were discharged alive from the hospitals, were considered for the analysis. The exclusion criteria included infants with a congenital malformation, chromosomal abnormalities, those who died before discharge from the hospital, and had a hospital stay less of than two weeks. GA estimation was done by a combination of last menstrual period, ultrasound and New Ballard Score assessment. Variables such as birth weight, discharge weight, estimated GA, clinical profile of the infants, corrected age and duration of hospital stay were analyzed.

Statistical analysis

Weight for GA and weight for corrected age z-scores were calculated using gender specific Fenton growth chart calculation spreadsheets.[19] SGA and EUGR was defined as weight for GA and weight at discharge for corrected age <-1.29 or less than the 10th percentile respectively. Statistical analyses were done using SPSS version 23 software. Descriptive statistics and binary logistic regression were performed with a significance level of 5% and with a 95% confidence interval (95% CI).

Patient and Public Involvement statement

Confidential: For Review Only

Study participants were not involved in the design of the study.

Ethical considerations

The study was conducted after ethical approval was obtained from Addis Ababa University College of health Sciences institutional review board (Ethics ID: AAUMF 03-008), and LMU Institutional Review Board (Ethics ID: 19-649). The parents of the infants were given adequate information and asked for informed consent prior to participation.

Results

Figure 1 shows the flow chart of recruitment of study subjects, including those who were excluded because they were discharged early, died in the hospital or had chromosomal abnormalities and congenital malformations. A total of 436 preterm infants were eligible for the analysis, 223(51%) were male. Nearly half 205 (47%) of the infants were very preterm (born at GA of 28 to 32 weeks) and 224 (51.4%) were very low birth weight (birth weight less than 1500gms). The rate of small for GA (SGA) among the study subjects was 42.4%, while 55.5% and 2.1% were appropriate for GA (AGA) and large for GA (LGA) respectively. The birth weight for GA Z-score, mean (SD) was -1.1 (1.0), while weight for corrected age Z-score mean (SD) at the time of discharge was -2.5 (1.1) (Table 1).

Nearly half of the infants, 214 (49.1%) had neonatal infections such as neonatal sepsis, pneumonia, meningitis and NEC, while 190 (43.6%), 111 (25.6%), 253 (58%), and 19 (4.4%) had respiratory distress syndrome, feeding problems, hypothermia and perinatal asphyxia

https://mc.manuscriptcentral.com/bmjpo

Confidential: For Review Only

respectively. The mean (SD) duration of hospital stay was 21.5 (5.1) days and the mean (SD) of corrected age at discharge was 35.4 (1.9) weeks.

The overall incidence of EUGR was 86.2%. Almost all (98.4%) of the infants born SGA had EUGR at discharge, while fewer of the LGA cases (22.2%) were classified as EUGR at the time of discharge from the hospital. Comparable rates of EUGR were observed across the infants' diagnoses.

Birth weight, weight for gestational age, and duration of hospital stay were found to be associated with the occurrence of EUGR (P-value <0.01) (Table 2). Variables associated with a statistically significant increased risk of EUGR on binary logistic regression include being SGA, very low birth weight, and duration of hospital stay greater than 21 days. VLBW infants had a 15-fold increased risk of developing EUGR at the time discharge from hospital than those who had birth weight greater than 1500gms (odds ratio = 15.2; 95% CI, 4.6-50.1) (Table 3).

Confidential: For Review Only

Table1. Clinical characteristics of the preterm infants

**Variables**	**Values**
Female /male ratio **(%)**	49/51
**GA (weeks), No. (%)**	
28 to32	205 (47.0)
32 to 34	148 (33.9)
35 to <37	83 (19.0)
**Birth weight (g), No. (%)**	
< 1000	28 (6.4)
1000 to <1500	196 (45.0)
1500 to <2000	164 (37.6)

≥2000	48 (11.0)
**Weight for gestational age, No. (%)**	
AGA	242 (55.5)
LGA	9 (2.1)
SGA	185 (42.4)
Weight for gestational age Z-score, mean (SD)	-1.1 (1.0)
Weight for corrected age, mean (SD)	-2.5 (1.1)
**Newborn major diagnosis, No. (%)***	
Neonatal infections	214 (49.1)
Respiratory distress syndrome	190 (43.6)
Feeding problems	111 (25.6)
Perinatal asphyxia	19 (4.4)
Hypothermia	253 (58.0)
Anemia	82 (18.8)
Total duration of Hospital stay, mean days SD)	21.5 (5.1)
Corrected age at discharge, mean weeks (SD)	35.4 (1.9)

* The percent does not add up to 100 since the infants may have had more than one diagnosis.

GA = Gestational age, AGA = Appropriate for gestational age, LGA= Large for gestational age,

SGA = Small for gestational age

Confidential: For Review Only

Table 2. Incidence of growth restriction (EUGR) at discharge by estimated gestational age and birth weight

**Variables**	**No.**	**No. of EUGR cases**	**Percent**
Overall incidence of EUGR	436	376	86.2
**Gender**			
Female	213	185	86.9
Male	223	191	85.6
**Birth weight category***			
<1000	28	27	96.4
1000-1499	196	183	93.4
1500-2000	164	130	79.3
>2000	48	36	75.0
**Weight for GA***			
AGA	242	192	79.3
LGA	9	2	22.2
SGA	185	182	98.4
**Major diagnosis of the preterm infants**			
Infection	214	184	86.0
Respiratory distress syndrome	190	162	85.3
Perinatal asphyxia	19	16	84.2
Feeding problems	131	111	84.7
Hypothermia	253	207	81.8
Anemia	82	67	81.7
**Duration of Hospital stay***			
14-21 days	224	179	79.9
>21 days	212	197	92.9

* P-value less than 0.01

EUGR = Extrauterine growth restriction, GA = Gestational age, AGA = Appropriate for gestational age, LGA= Large for gestational age, SGA = Small for gestational age

Confidential: For Review Only

Table 3. Variables associated with extrauterine growth restriction

Variables	Total No.	EUGR No. (%)	P- value	AOR	95 % C.I	
					lower	upper
**Birth weight**						
≥ 1500 grams	212	166 (78.3)	-	-	-	-
<1500 grams	224	210 (93.8)	.00	15.2	4.6	50.1
**Weight for GA**						
Non-SGA	251	194 (77.3)	-	-	-	-
SGA	185	182 (98.4)	.03	2.2	1.1	4.3
**Hospital stay**						
<21 days	224	179 (79.9)	-	-	-	-
≥21 days	212	197 (92.9)	.00	2.7	1.4	5.3

AOR= adjusted odds ratio, GA= Gestational age, EUGR = Extrauterine growth restriction, SGA = Small for gestational age

Discussion

The SIP study had a very high mortality rate (29%) among hospital admitted preterm infants.[17] The current follow-up study revealed that most (86.2%) of the infants who survived the immediate complications were discharged with EUGR and associated severe caloric and protein deficits.[7] Establishing adequate dietary intakes in preterm infants is a very common problem in NICUs, however optimal nutrition is critically important to insure survival, normal growth and development in subsequent years.[20, 21]

The incidence of EUGR observed in this study was comparable to the 89% EUGR rate in extremely low birth weight infants (Birthweight less than 1000gm) reported by Dusick et al. from the USA,[22] however only 6.4% of the study population in the current study were extremely low birth weight. The rate of EUGR in this study was much higher than that reported from China by Shan et al,; Lima PA et al from Brazil and Clark et al. from the USA, 56.8%,[23]

Confidential: For Review Only

26%,[6]28%,[5] respectively. Nearly half the preterm infants in the current study had a birth weight greater than 1500 grams, while the other studies included mainly very low birth weight infants and those with extreme prematurity. In addition, our study did not include infants with a GA less than 28 weeks. Thus, our study shows a rate of EUGR that was unacceptably high in later GA preterm infants.

The mean Z- score of birth weight and weight at discharge in this study was significantly lower than the averages reported in other literature.[6, 24] Shan, H.M., et al. have shown risk factors related to EUGR, such as male gender, low gestational age at birth, low birth weight, and long length of hospital stay.[23] Similarly, we found an increased risk of EUGR with very low birth weight, prolonged duration of hospital stay and SGA (Table 3). This finding is similar to a report of Sakurai, M., et al from Japan, in which they found lower GA, intrauterine growth restriction, severe chronic lung disease, and poor nutrition as relevant risk factors associated with EUGR.[25] Preterm infants born SGA have higher risk of morbidity and mortality compared to AGA preterm infants.[6, 26]The rate of EUGR was higher (92.9%) in infants who stayed in the hospital for more than 21 days. This is likely explained by the severity of the infant morbidities and the inadequacy of nutritional support provided in the NICUs.

Aggressive nutritional support has been shown to promote growth without increased risk of adverse effects.[27]With optimal nutrition, postnatal growth failure could be prevented and extrauterine weight gain can be achieved similar to fetuses of same GA.[28] A combination of parenteral nutrition, early advancement of enteral feeding and fortification of human milk are the current standards of care in developed countries.[3, 29] These interventions are often not available in low-income countries. Under-nutrition experienced in infancy is known to impair

Confidential: For Review Only

cognitive function, school achievement, and results in increased risk of behavioral problems later in life.[30]

Conclusion

The high incidence of EUGR observed in this study indicates that the nutritional support of the preterm infants was insufficient. The risk of developing EUGR was higher in preterm infants who had lower GA, very low birth weight, prolonged duration of hospital stay and SGA. Much attention needs to be given to improve preterm nutrition in low-income countries. Country infant feeding guidelines need revision based on recent evidence to improve preterm nutrition. Regular monitoring of nutritional status and individualized timely nutritional intervention has to be the standard of care of preterm infants in the NICUs.

Acknowledgments: We would like thank Bill and Melinda Gates foundation for funding the SIP study. Our appreciation goes to all the families who participated in this study and those who contributed for data collection and completion of the study.

Author contributions: The primary study from which the data was extracted "Causes of Illness and Death of Preterm Infants in Ethiopia (SIP)" was conceptualized and designed by AKN and LMM; data collection was monitored by LMM, BW, EMM, AM and RLG. NWG analyzed the data and drafted the manuscript and RLG, AKN, EMM, AM, BW, MS, OG and LMM contributed in writing the manuscript. All authors have revised the work critically and approved the final manuscript as submitted. All the authors have contributed for the manuscript, and approved the final version.

Confidential: For Review Only

Data Availability: The data associated with this paper is available from the corresponding author upon reasonable request.

Research funding: This study was supported by Bill and Melinda Gates foundation.

Competing interests: None of the authors had competing of interests.

References

1. Howson CP, Kinney MV, McDougall L, Lawn JE. Born too soon: preterm birth matters. *Reprod. Health* 2013;10:S1.
2. Embleton NE, Pang N, Cooke RJ. Postnatal malnutrition and growth retardation: an inevitable consequence of current recommendations in preterm infants? *Pediatrics* 2001;107:270 –3.
3. Ziegler EE. Meeting the nutritional needs of the low-birth-weight infant. *Ann Nutr Meta* 2011;58(Suppl. 1):8–18.
4. Lunde D. Extrauterine Growth Restriction: What is the Evidence for Better Nutritional Practices in the Neonatal Intensive Care Unit? *Newborn Infant Nurs Rev* 2014;14:92–98.
5. Clark RH, Thomas P, Peabody J. Extrauterine growth restriction remains a serious problem in prematurely born neonates. *Pediatrics* 2003;111:986–90.
6. Lima PA, de Carvalho M, da Costa AC, Moreira ME. Variables associated with extra uterine growth restriction in very low birth weight infants. *J Pediatr (Rio J)*2014;90:22–7.
7. Su BH. Optimizing nutrition in preterm infants. *Pediatr Neonatol* 2014;55:5–13.

Confidential: For Review Only

8. Schanler RJ, Shulman RJ, Lau C. Feeding strategies for premature infants: beneficial outcomes of feeding fortified human milk versus preterm formula. *Pediatrics* 1999;103:1150–7.
9. Clark RH, Wagner CL, Merritt RJ, Bloom BT, Neu J, Young TE, Clark DA. Nutrition in the neonatal intensive care unit: how do we reduce the incidence of extrauterine growth restriction? *J. Perinatol* 2003;23:337–44.
10. Leaf A, Dorling J, Kempley S, et al. Early or delayed enteral feeding for preterm growth-restricted infants: a randomized trial. *Pediatrics* 2012;129:e1260–8.
11. Isaacson L. Steps to successfully breastfeed the premature infant. *Neonatal Netw* 2006;25:77–86.
12. Kumar RK, Singhal A, Vaidya U, Banerjee S, Anwar F, Rao S. Optimizing nutrition in preterm low birth weight infants—Consensus summary. *fnut* 2017;4:20.
13. Parker LA, Neu J, Torrazza RM, Li Y. Scientifically based strategies for enteral feeding in premature infants. *Neoreviews* 2013;14:e350–9.
14. Ong KK, Kennedy K, Castañeda-Gutiérrez E, et al. Postnatal growth in preterm infants and later health outcomes: a systematic review. *Acta Paediatr* 2015;104:974–986.
15. Blencowe H, Cousens S, Chou D, et al. Born too soon: the global epidemiology of 15 million preterm births. *Reprod. Health* 2013;10:S2.
16. Muhe LM, McClure EM, Mekasha A, et al. A prospective study of causes of illness and death in preterm infants in Ethiopia: the SIP study protocol. *Reprod. Health* 2018;15:116.
17. Muhe LM, McClure EM, Nigussie AK, et al. Major causes of death in preterm infants in selected hospitals in Ethiopia (SIP): a prospective, cross-sectional, observational study. *Lancet Glob. Health* 2019 ;7:e1130–8.

https://mc.manuscriptcentral.com/bmjpo
Confidential: For Review Only

18. Federal Ministry of Health of Ethiopia. Neonatal Intensive Care Unit (NICU) management protocol. Addis Ababa: Federal Ministry of Health of Ethiopia, 2014.

19. Fenton TR, Kim JH. A systematic review and meta-analysis to revise the Fenton growth chart for preterm infants. *BMC Pediatr* 2013;13:59.

20. Cooke R. Postnatal growth in preterm infants: have we got it right? *J. Perinatol* 2005;25:S12–4.

21. Carlson SJ, Ziegler EE. Nutrient intakes and growth of very low birth weight infants. *J Perinatol* 1998;18:252–8.

22. Dusick AM, Poindexter BB, Ehrenkranz RA, Lemons JA. Growth failure in the preterm infant: can we catch up? *Semin. Perinatol* 2003;27:302–310.

23. Shan HM, Cai W, Cao Y, Fang BH, Feng Y. Extrauterine growth retardation in premature infants in Shanghai: a multicenter retrospective review. *Eur J Pediatr* 2009;168:1055.

24. Rochow N, Landau-Crangle E, So HY, et al. Z-score differences based on cross-sectional growth charts do not reflect the growth rate of very low birth weight infants. *PloS one* 2019;14(5).

25. Sakurai M, Itabashi K, Sato Y, Hibino S, Mizuno K. Extrauterine growth restriction in preterm infants of gestational age≤ 32 weeks. *Pediatr. Int* 2008;50:70–75.

26. Simchen MJ, Beiner ME, Strauss-Liviathan N, et al. Neonatal outcome in growth-restricted versus appropriately grown preterm infants. *AM J PERINAT* 2000;17:187192.

27. Hay WW. Aggressive nutrition of the preterm infant. *Curr. Pediatr. Rep* 2013;1:229–239.

Confidential: For Review Only

28. Andrews ET, Ashton JJ, Pearson F, Beattie RM, Johnson MJ. Early postnatal growth failure in preterm infants is not inevitable. *Arch. Dis* 2019;104:F235–41.

29. Agostoni C, Buonocore G, Carnielli VP, et al. Enteral nutrient supply for preterm infants: commentary from the European Society of Paediatric Gastroenterology, Hepatology and Nutrition Committee on Nutrition. *J. Pediatr. Gastroenterol. Nutr* 2010;50:85–91.

30. Prado EL, Dewey KG. Nutrition and brain development in early life. *Nutr. Rev* 2014;72:267–284.

Confidential: For Review Only

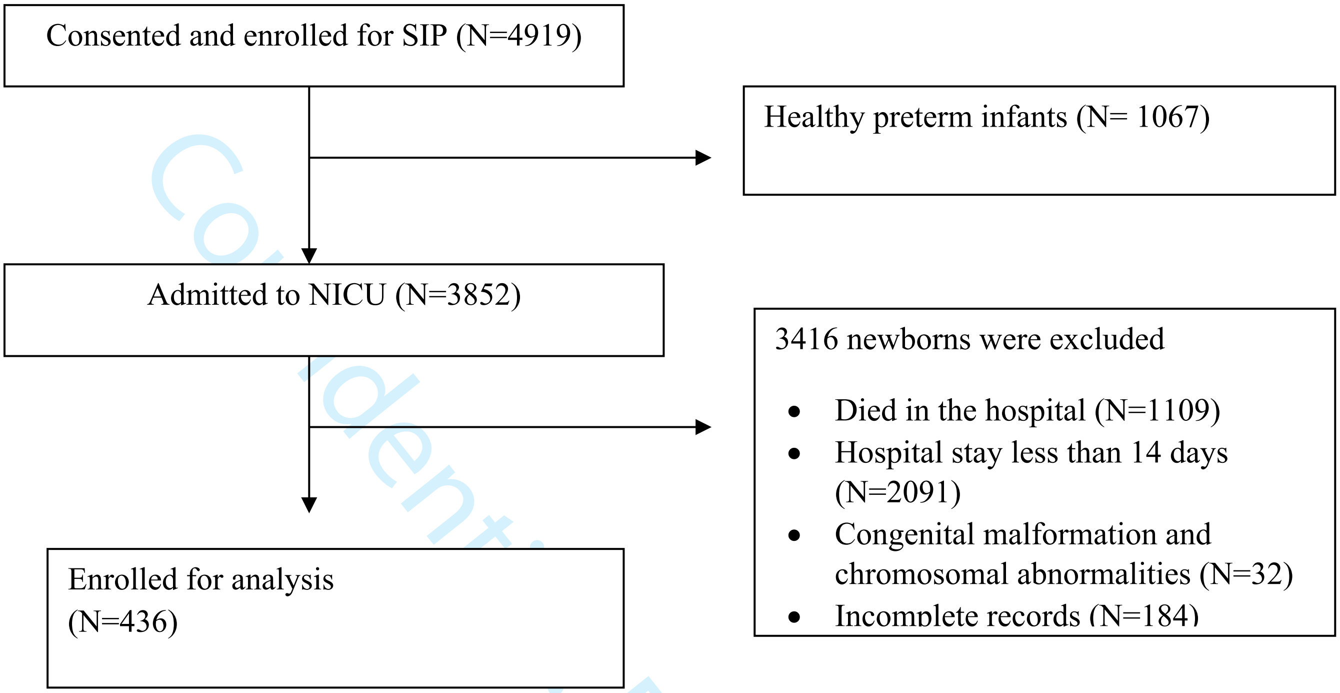

Figure 1. Flow chart of study subjects included in the analysis.

Confidential: For Review Only

BMJ Paediatrics Open

Incidence and associated factors of extrauterine growth restriction (EUGR) in preterm infants, a cross-sectional study in selected NICUs in Ethiopia

Journal:	*BMJ Paediatrics Open*
Manuscript ID	bmjpo-2020-000765.R1
Article Type:	Original research
Date Submitted by the Author:	25-Jul-2020

Complete List of Authors:	Gidi, Netsanet; Jimma University College of Public Health and Medical Sciences, Pediatric and child health<br>Goldenberg , Robert L. ; Columbia University<br>Nigussie, Assaye K. ; Bill and Melinda Gates Foundation<br>McClure, Elizabeth; RTI International<br>Mekasha, Amha; Addis Ababa University College of Health Sciences<br>Worku, Bogale; Ethiopian Pediatric Society<br>Siebeck, Matthias; University Hospital, LMU Munich, Germany, Institute for Medical Education<br>Genzel-Boroviczeny, Orsolya; Ludwig Maximilian University of Munich , Dr. von Hauner University Children's Hospital<br>Muhe, Lulu M; Addis Ababa University College of Health Sciences

Keywords:	Neonatology

SCHOLARONE™
Manuscripts

I, the Submitting Author has the right to grant and does grant on behalf of all authors of the Work (as defined in the below author licence), an exclusive licence and/or a non-exclusive licence for contributions from authors who are: i) UK Crown employees; ii) where BMJ has agreed a CC-BY licence shall apply, and/or iii) in accordance with the terms applicable for US Federal Government officers or employees acting as part of their official duties; on a worldwide, perpetual, irrevocable, royalty-free basis to BMJ Publishing Group Ltd ("BMJ") its licensees and where the relevant Journal is co-owned by BMJ to the co-owners of the Journal, to publish the Work in this journal and any other BMJ products and to exploit all rights, as set out in our licence.

The Submitting Author accepts and understands that any supply made under these terms is made by BMJ to the Submitting Author unless you are acting as an employee on behalf of your employer or a postgraduate student of an affiliated institution which is paying any applicable article publishing charge ("APC") for Open Access articles. Where the Submitting Author wishes to make the Work available on an Open Access basis (and intends to pay the relevant APC), the terms of reuse of such Open Access shall be governed by a Creative Commons licence – details of these licences and which Creative Commons licence will apply to this Work are set out in our licence referred to above.

Other than as permitted in any relevant BMJ Author's Self Archiving Policies, I confirm this Work has not been accepted for publication elsewhere, is not being considered for publication elsewhere and does not duplicate material already published. I confirm all authors consent to publication of this Work and authorise the granting of this licence.

Confidential: For Review Only

Title: Incidence and associated factors of extrauterine growth restriction (EUGR) in preterm infants, a cross-sectional study in selected NICUs in Ethiopia

Authors: Dr. Netsanet Workneh Gidi[a,i], Dr. Robert L. Goldenberg[b], Prof. Assaye K. Nigussie[c], Dr. Elizabeth M. McClure[d], Prof. Amha Mekasha[e], Prof. Bogale Worku[f], Prof. Dr. Matthias Siebeck[g], Prof. Dr. Orsolya Genzel-Boroviczény[h], Prof. Lulu M. Muhe[e]

Affiliations

[a] Institute of Health Sciences, Jimma University, Jimma, Ethiopia

[b] Columbia University, New York, NY, USA

[c] Bill and Melinda Gates Foundation, Seattle, WA, USA

[d] RTI International, 3040 Cornwallis Rd., Durham, NC, 27709, USA

[e] College of Health Sciences, Addis Ababa University, Addis Ababa, Ethiopia

[f] Ethiopian Pediatric Society, Addis Ababa, Ethiopia

[g] Institute for Medical Education, University Hospital, LMU Munich, Germany

[h] Dr von Hauner Childrens´Hospital, University Hospital, LMU Munich, Germany

[i] Center for International Health, University Hospital, LMU Munich, Germany

https://mc.manuscriptcentral.com/bmjpo

Corresponding author: Netsanet Workneh Gidi (MD, DTM&H)

Associate professor of pediatrics and child health

College of public health and medical sciences, Jimma University, Jimma, Ethiopia

PhD candidate at CIHLMU, Munich, Germany

Phone: +251 917762109

Email: konetsanet@gmail.com, or Netsanet.Workneh@lrz.uni-muenchen.de

Confidential: For Review Only

Abstract

Background: Preterm infants have high risk of developing growth restriction and long term complications. Enteral feeding is often delayed in neonatal intensive care units (NICUs) for the fear of feeding intolerance and the associated necrotizing enterocolitis, and recent advances in nutritional support are unavailable in low income countries.

Objective: The aim of this study was to assess the incidence and associated factors of extrauterine growth restriction (EUGR) among preterm infants in selected NICUs in Ethiopia.

Method: This was a cross-sectional study involving a sub-group analysis of preterm infants admitted to hospitals, from a multicenter descriptive study of cause of illness and death in preterm infants in Ethiopia, conducted from 2016 to 2018. EUGR was defined as weight at discharge z-scores <-1.29 for corrected age. Clinical profiles of the infants were analyzed for associated factors. SPSS version 23 software was used for analysis with a significance level of 5% and 95% confidence interval.

Result: From 436 preterm infants included in the analysis, 223(51%) were male, 224 (51.4%) very low birth weight (VLBW) and 185(42.4%) small for gestational age (SGA). The mean (SD) of weight for corrected age Z-score at the time of discharge was -2.5 (1.1). The incidence of EUGR was 86.2%. Infants who were SGA, VLBW, and longer hospital stay over 21 days had increased risk of growth restriction (p-value <0.01). SGA infants had a 15-fold higher risk of developing EUGR at the time of discharge from hospital than those who were appropriate or large for gestational age (OR (95% CI) = 15.2 (4.6,50.1).

Conclusion: The majority of the infants had EUGR at the time of discharge from the hospital, which indicates suboptimal nutrition. Revision of national guidelines for preterm infants feeding and improvement in clinical practice is highly required.

Key words: EUGR; SGA; VLBW, preterm nutrition

What is known about the subject?

- Preterm infants' growth is expected to be similar to that of the intrauterine fetus, however small preterm infants often develop extrauterine growth restriction (EUGR).
- EUGR is associated with increased risk of post neonatal mortality and long-term morbidities such as adverse metabolic and neurodevelopmental outcomes in subsequent years.
- There is paucity of data regarding preterm nutrition in low and middle-income countries; parenteral nutrition and use of human milk fortifiers are often unavailable.

Confidential: For Review Only

What this study adds?

- Neonatal mortality rate of hospital admitted preterm infants in Ethiopia is 29%, 86.2% of the infants who survived the immediate complications were discharged with EUGR.
- The high incidence of EUGR indicates insufficient nutritional support of the preterm infants.
- Infants with very low birth weight, hospital stay over 21 days and small for gestational age had increased risk of developing EUGR.

Introduction

Complications of preterm births are the leading causes of newborn deaths worldwide. Survivors of preterm birth are at increased risk of adverse metabolic and neurodevelopmental long-term outcomes.[1] Ideally, growth of the preterm infant is expected to be similar to that of the intrauterine fetus at the same gestational age (GA) once birth weight has been regained. However, attaining that goal requires optimal nutritional support to address the increased needs of nutrients for catch-up growth.[2, 3] Extrauterine growth restriction (EUGR) is a severe nutritional deficit during the first weeks after birth, commonly seen in small preterm infants.[4, 5] Factors associated with EUGR reported from developed countries include caloric and protein deficits, intrauterine growth restriction (IUGR), neonatal morbidities and the need for prolonged hospital stay.[6] EUGR is commonly defined as a growth measurement that is < 10th percentile of the predicted value at the time of hospital discharge.[5]

Improved nutritional support may decrease the rate of EUGR. This support may include early administration of parenteral nutrition, use of human milk fortifiers, and preterm formula when

Confidential: For Review Only

mother's milk is unavailable.[7, 8] Identifying infants at risk of growth failure by monitoring weight and nutritional intake should guide clinicians to increase nutritional support that is individualized according to the need of the preterm infant.[9] Many mothers of preterm infants need support to produce and express enough milk as the babies are often too weak to suckle.[10,11] Recent evidence indicates that early, fast or continuous enteral feeding results in better neonatal outcomes compared to late, slow or intermittent feeding.[10, 12] However, clinicians in many neonatal intensive care units (NICUs) often delay enteral feeding for the fear of feeding intolerance and the associated necrotizing enterocolitis (NEC).[13]

For LMICs WHO guidelines on feeding stable low birth weight infants whose birthweight is greater than 1000gm recommend feeding mother's own milk starting from the first day. Those who cannot be fed mother's own milk should be fed donor human milk (when available); if this is not possible standard infant formula has to be given, those who fail to gain weight despite adequate feeding with standard infant formula should be given preterm infant formula. And VLBW infants who fail to gain weight despite adequate breast milk feeding should be given human-milk fortifiers.[14] In Ethiopian neonatal guideline, mothers breast milk is the only option recommended for feeding preterm infants; however the use of donors milk, standard infant formula milk, preterm formula milk and human milk fortifiers were not considered as options where indicated.[15] EUGR is associated with long-term morbidities such as adverse neurodevelopmental outcomes in subsequent years. Hence, assessment of the magnitude of the problem and recognizing associated factors should help to identify and manage preterm infants at risk of growth restriction and consequently improve long-term outcomes.[16] The burden of preterm birth is increasing worldwide, and the highest average rate of preterm birth occurs in low-income countries (11.8%).[17] However, there is paucity of data regarding preterm nutrition

Confidential: For Review Only

and EUGR in low and middle-income countries, most of the literatures in this area are reported from high-income countries. Thus, the aim of this study is to assess the incidence and associated factors of EUGR in preterm infants in 5 NICUs in Ethiopia.

Methods

Data Source

This is a cross-sectional study involving the analysis of a sub-group of 436 preterm infants from a multicenter descriptive study conducted in five selected hospitals, "Study of causes of illness and death in preterm infants (SIP)". The primary study and methodology papers have been previously published.[18, 19] The hospital practice of neonatal care was based on a national neonatal guideline. Stable preterm infants are fed on mothers own breast milk. For infants weighing < 1.5 kg at birth, starting expressed breast milk 10 ml/kg per day and increasing the amount by 20ml/kg/day according to the infants' condition until full volume feeding is achieved. The goal is to achieve, volume: 140 – 150 ml/kg/day & calorie: 110 – 120 kcal/kg/day. Other nutritional support methods, such as the use of donor milk, parenteral nutrition and breast milk fortification were not available.[15]

Preterm infants with a GA of 28 to 36 weeks, who were discharged alive from the hospitals, were considered for the analysis. The exclusion criteria included infants with a congenital malformation, chromosomal abnormalities; those who died before discharge from the hospital, and had a hospital stay less of than two weeks. GA estimation was done by a combination of last menstrual period, ultrasound and New Ballard Score assessment. Variables such as birth weight, discharge weight, estimated GA, clinical profile of the infants, corrected age and duration of hospital stay were analyzed.

Confidential: For Review Only

Statistical analysis

Weight for GA and weight for corrected age z-scores were calculated using gender specific Fenton growth chart calculation spreadsheets.[20] SGA and EUGR was defined as weight for GA and weight at discharge for corrected age <-1.29 or less than the 10th percentile respectively.

Statistical analyses were done using SPSS version 23 software. Following descriptive analysis, the chi-square test was used to check the cell count adequacy before performing univariate logistic regression. Factors that could be associated with the dependent variables were identified from univariate logistic regression (P-value < 0.2). Stepwise multivariate logistic regression was performed to identify independent risk factors for EUGR with significance level of 5% and 95% confidence interval (95% CI)."

Patient and Public Involvement statement

Study participants were not involved in the design of the study.

Ethical considerations

The study was conducted after ethical approval was obtained from Addis Ababa University College of health Sciences institutional review board (Ethics ID: AAUMF 03-008), and LMU Institutional Review Board (Ethics ID: 19-649). The parents of the infants were given adequate information and asked for informed consent prior to participation.

Results

Figure 1 shows the flow chart of recruitment of study subjects, including those who were excluded because they were discharged early, died in the hospital or had chromosomal

Confidential: For Review Only

abnormalities and congenital malformations. A total of 436 preterm infants were eligible for the analysis, 223(51%) were male. Nearly half 205 (47%) of the infants were very preterm (born at GA of 28 to 32 weeks) and 224 (51.4%) were very low birth weight (birth weight less than 1500gms). The rate of small for GA (SGA) among the study subjects was 42.4%, while 55.5% and 2.1% were appropriate for GA (AGA) and large for GA (LGA) respectively. The birth weight for GA Z-score, mean (SD) was -1.1 (1.0), while weight for corrected age Z-score mean (SD) at the time of discharge was -2.5 (1.1) (Table 1).

Nearly half of the infants, 214 (49.1%) had neonatal infections such as neonatal sepsis, pneumonia, meningitis and NEC, while 190 (43.6%), 111 (25.6%), 253 (58%), and 19 (4.4%) had respiratory distress syndrome, feeding problems, hypothermia and perinatal asphyxia respectively. The mean (SD) duration of hospital stay was 21.5 (5.1) days and the mean (SD) of corrected age at discharge was 35.4 (1.9) weeks.

The overall incidence of EUGR was 86.2%. Almost all (98.4%) of the infants born SGA had EUGR at discharge, while fewer of the LGA cases (22.2%) were classified as EUGR at the time of discharge from the hospital. Comparable rates of EUGR were observed across the infants' major diagnoses. Birth weight, weight for gestational age, and duration of hospital stay were found to be associated with the occurrence of EUGR (p-value <0.01) (Table 2). Variables associated with a statistically significant increased risk of EUGR on univariate logistic regression include being SGA, very low birth weight, and duration of hospital stay over 21days. Similarly, on stepwise multivariate logistic regression, SGA, very low birth weight, and longer hospital stay over 21days were found to be independent risk factors for EUGR. SGA infants had a 15-fold increased risk of developing EUGR at the time discharge from hospital than those who were appropriate or large for gestational age (OR (95% CI) = 15.2;, (4.6,50.1) (Table 3).

Confidential: For Review Only

Table1. Clinical characteristics of the preterm infants

Variables	Values
Female /male ratio **(%)**	49/51
**GA (weeks), No. (%)**	
28 to32	205 (47.0)
32 to 34	148 (33.9)
35 to <37	83 (19.0)
**Birth weight (g), No. (%)**	
< 1000	28 (6.4)
1000 to 1500	196 (45.0)
1500 to 2000	164 (37.6)
≥2000	48 (11.0)
**Weight for gestational age, No. (%)**	
AGA	242 (55.5)
LGA	9 (2.1)
SGA	185 (42.4)
**Pregnancy**	
Singleton	260 (59.6)
Twins	166 (38.1)
Triplets	10 (2.3)
Weight for gestational age Z-score at birth, mean (SD)	-1.1 (1.0)
Weight for corrected age Z-score at discharge, mean (SD)	-2.5 (1.1)
**Newborn major diagnosis, No. (%)***	
Neonatal infections	214 (49.1)
Respiratory distress syndrome	190 (43.6)
Feeding problems	111 (25.6)
Perinatal asphyxia	19 (4.4)
Hypothermia	253 (58.0)
Anemia	82 (18.8)
Total duration of Hospital stay, mean days SD)	21.5 (5.1)
Corrected age at discharge, week, mean(SD)	35.4 (1.9)

* The percent does not add up to 100 since the infants may have had more than one diagnosis.

GA = Gestational age, AGA = Appropriate for gestational age, LGA= Large for gestational age,

SGA = Small for gestational age

Confidential: For Review Only

Table 2. Univariate logistic regression, factors associated with extrauterine growth restriction (EUGR)

**Variables**	**Total No.**	**EUGR cases No. (%)**	**p value**	**OR (95%CI)**
Overall incidence of EUGR	436	376(86.2)	-	-
**Gender**				
Female	213	191(86.9)	-	-
Male	223	185(85,6)	0.71	0.9(0.5,1.6)
**Birth weight**				
<1500	224	**210(93.8)**	**0<.001**	**4.9 (2.2,11.1)**
>1500	212	16678.3)	-	-
**Weight for GA**				
AGA and LGA	251	194 (77.3)	-	-
SGA	185	**182 (98.4)**	**0<.001**	**17.8 (5.4, 57.9)**
**Pregnancy**				
Singleton	260	226	**-**	**-**
Twins and triplets	176	150	0.61	1.2 (0.7, 2.0)
**Major diagnosis of the preterm infants**				
Infection	214	184(86.0)	0.87	1.0 (0.55, 1.65)
Respiratory distress syndrome	190	162(85.3)	0.60	0.8 (0.5,1.5)
Perinatal asphyxia	19	16(84.2)	0.79	0.8 (0.2, 2.9)
Feeding problems	131	111(84.7)	0.55	0.8 (0.6,1.5)
Anemia	82	67(81.7)	0.18	0.7 (0.3, 1.2)
**Duration of Hospital stay**				
14-21 days	224	179 (79.9)	-	-
>21 days	212	**197 (92.9)**	**0<.001**	**3.3 (1.8,6.1)**

EUGR = Extrauterine growth restriction, GA = Gestational age, AGA = Appropriate for gestational age, LGA= Large for gestational age, SGA = Small for gestational age

Confidential: For Review Only

Table 3. Multivariate logistic regression analysis, independent risk factors of extrauterine growth restriction

Variables	P-value	AOR (95% CI)
**Birth weight**		
Non-SGA	-	-
SGA	<0.001	15.2 (4.6,50.1)
**Weight for GA**		
≥ 1500 grams	-	-
<1500 grams	0.03	2.2 (1.1,4.3)
**Duration of hospital stay**		
<21 days	-	-
≥21 days	<0.001	2.7 (1.4,5.3)

AOR= adjusted odds ratio, GA= Gestational age, EUGR = Extrauterine growth restriction, SGA = Small for gestational age

Discussion

The SIP study has showed a very high mortality rate (29%) among hospital admitted preterm infants.[19] The current follow-up study revealed that most (86.2%) of the infants who survived the immediate complications were discharged with EUGR and associated severe caloric and protein deficits. Establishing adequate dietary intakes in preterm infants is a very common problem in NICUs; however optimal nutrition is critically important to insure survival, normal growth and development in subsequent years.[21, 22]

The incidence of EUGR observed in this study was comparable to the 89% EUGR rate in extremely low birth weight infants (Birthweight less than 1000gm) reported by Dusick et al. from the USA,[23] however only 6.4% of the study population in the current study were extremely low birth weight. The rate of EUGR in this study was much higher than that reported from China by Shan et al,; Lima PA et al from Brazil and Clark et al. from the USA, 56.8%,[24]

Confidential: For Review Only

26%,[6]28%,[5] respectively. Nearly half the preterm infants in the current study had a birth weight greater than 1500 grams, while the other studies included mainly very low birth weight infants and those with extreme prematurity. In addition, our study did not include infants with a GA less than 28 weeks. Thus, our study shows a rate of EUGR that was unacceptably high in higher GA preterm infants.

The mean Z- score of birth weight and weight at discharge in this study was significantly lower than the averages reported in other literature.[6, 25] Shan, H.M., et al. have shown risk factors related to EUGR, such as male gender, low gestational age at birth, low birth weight, and long length of hospital stay.[24] In this study, we found increased risk of EUGR in infants who were SGA, very low birth weight, and hospitalized over 21days (Table 3). Sakurai, M., et al from Japan have also reported lower GA, intrauterine growth restriction, severe chronic lung disease, and poor nutrition as relevant risk factors associated with EUGR, the SGA infants in our study are likely to have had intrauterine growth restriction, but none of the comorbidities the infants had was associated with increased risk EUGR .[26] Generally, preterm infants born SGA have higher risk of morbidity and mortality compared to AGA preterm infants.[6, 27] The rate of EUGR was higher (92.9%) in infants who stayed in the hospital over 21 days. This is probably due to the severity of the infants' morbidities and the inadequacy of nutritional support provided in the NICUs.

Aggressive nutritional support has been shown to promote growth without increased risk of adverse effects.[28] With optimal nutrition, postnatal growth failure could be prevented and extrauterine weight gain can be achieved similar to fetuses of same GA.[29] A combination of parenteral nutrition, early advancement of enteral feeding and fortification of human milk are the

Confidential: For Review Only

current standards of care in developed countries.[3, 30] These interventions are often not available in low-income countries. Under-nutrition experienced in infancy is known to impair cognitive function, school achievement, and results in increased risk of behavioral problems later in life.[31]

This study has several limitations, the mean corrected age at discharge was 35.4 weeks, and follow up at around 40 weeks could have possibly shown catch up growth. We used similar definition of EUGR for all infants in the study, SGA infants' growth velocity was not considered for diagnosis EUGR. Nutritional data, maternal conditions, and delivery relating factors were not assessed as risk factors. This was a cross sectional study design, the main aim was to assess the incidence of EUGR, associated factors were analysed with the available data. Further study is required to identify all the predictors of EUGR.

Conclusion

The high incidence of EUGR observed in this study indicates that the nutritional support of the preterm infants was insufficient. The risk of developing EUGR was higher in preterm infants who were very low birth weight, SGA and hospitalized for over 21 days . Much attention needs to be given to improve preterm nutrition in low-income countries. Country infant feeding guidelines need revision based on recent evidences to improve preterm nutrition. Regular monitoring of nutritional status and individualized timely nutritional intervention has to be the standard of care of preterm infants in the NICUs.

Acknowledgments: We would like thank Bill and Melinda Gates foundation for funding the SIP study. Our appreciation goes to all the families who participated in this study and those who contributed for data collection and completion of the study.

Confidential: For Review Only

Author contributions: The primary study from which the data was extracted "Causes of Illness and Death of Preterm Infants in Ethiopia (SIP)" was conceptualized and designed by AKN and LMM; data collection was monitored by LMM, BW, EMM, AM and RLG. NWG analyzed the data and drafted the manuscript and RLG, AKN, EMM, AM, BW, MS, OG and LMM contributed in writing the manuscript. All authors have revised the work critically and approved the final manuscript as submitted. All the authors have contributed for the manuscript, and approved the final version.

Data Availability: The data associated with this paper is available from the corresponding author upon reasonable request.

Research funding: This study was supported by Bill and Melinda Gates foundation, grant number: OPP1136965.

Competing interests: None of the authors had competing of interests.

References

1. Howson CP, Kinney MV, McDougall L, Lawn JE. Born too soon: preterm birth matters. *Reprod. Health* 2013;10:S1.
2. Embleton NE, Pang N, Cooke RJ. Postnatal malnutrition and growth retardation: an inevitable consequence of current recommendations in preterm infants? *Pediatrics* 2001;107:270 –3.
3. Ziegler EE. Meeting the nutritional needs of the low-birth-weight infant. *Ann Nutr Meta* 2011;58(Suppl. 1):8–18.

Confidential: For Review Only

4. Lunde D. Extrauterine Growth Restriction: What is the Evidence for Better Nutritional Practices in the Neonatal Intensive Care Unit? *Newborn Infant Nurs Rev* 2014;14:92–98.
5. Clark RH, Thomas P, Peabody J. Extrauterine growth restriction remains a serious problem in prematurely born neonates. *Pediatrics* 2003;111:986–90.
6. Lima PA, de Carvalho M, da Costa AC, Moreira ME. Variables associated with extra uterine growth restriction in very low birth weight infants. *J Pediatr (Rio J)*2014;90:22–7.
7. Su BH. Optimizing nutrition in preterm infants. *Pediatr Neonatol* 2014;55:5–13.
8. Schanler RJ, Shulman RJ, Lau C. Feeding strategies for premature infants: beneficial outcomes of feeding fortified human milk versus preterm formula. *Pediatrics* 1999;103:1150–7.
9. Clark RH, Wagner CL, Merritt RJ, Bloom BT, Neu J, Young TE, Clark DA. Nutrition in the neonatal intensive care unit: how do we reduce the incidence of extrauterine growth restriction? *J. Perinatol* 2003;23:337–44.
10. Leaf A, Dorling J, Kempley S, et al. Early or delayed enteral feeding for preterm growth-restricted infants: a randomized trial. *Pediatrics* 2012;129:e1260–8.
11. Isaacson L. Steps to successfully breastfeed the premature infant. *Neonatal Netw* 2006;25:77–86.
12. Kumar RK, Singhal A, Vaidya U, Banerjee S, Anwar F, Rao S. Optimizing nutrition in preterm low birth weight infants—Consensus summary. *fnut* 2017;4:20.
13. Parker LA, Neu J, Torrazza RM, Li Y. Scientifically based strategies for enteral feeding in premature infants. *Neoreviews* 2013;14:e350–9.

Confidential: For Review Only

14. WHO. Guidelines on optimal feeding of low birth-weight infants in low- and middle-income countries. Geneva, World Health Organization; 2011 (http://www.who.int/maternal_child_adolescent/documents/infant_feeding_low_bw/en/).

15. Federal Ministry of Health of Ethiopia. Neonatal Intensive Care Unit (NICU) management protocol. Addis Ababa: Federal Ministry of Health of Ethiopia, 2014.

16. Ong KK, Kennedy K, Castañeda-Gutiérrez E, et al. Postnatal growth in preterm infants and later health outcomes: a systematic review. *Acta Paediatr* 2015;104:974–986.

17. Blencowe H, Cousens S, Chou D, et al. Born too soon: the global epidemiology of 15 million preterm births. *Reprod. Health* 2013;10:S2.

18. Muhe LM, McClure EM, Mekasha A, et al. A prospective study of causes of illness and death in preterm infants in Ethiopia: the SIP study protocol. *Reprod. Health* 2018;15:116.

19. Muhe LM, McClure EM, Nigussie AK, et al. Major causes of death in preterm infants in selected hospitals in Ethiopia (SIP): a prospective, cross-sectionl, observational study. *Lancet Glob. Health* 2019 ;7:e1130–8.

20. Fenton TR, Kim JH. A systematic review and meta-analysis to revise the Fenton growth chart for preterm infants. *BMC Pediatr* 2013;13:59.

21. Cooke R. Postnatal growth in preterm infants: have we got it right? *J. Perinatol* 2005;25:S12–4.

22. Carlson SJ, Ziegler EE. Nutrient intakes and growth of very low birth weight infants. *J Perinatol* 1998;18:252–8.

23. Dusick AM, Poindexter BB, Ehrenkranz RA, Lemons JA. Growth failure in the preterm infant: can we catch up? *Semin. Perinatol* 2003;27:302–310.

Confidential: For Review Only

24. Shan HM, Cai W, Cao Y, Fang BH, Feng Y. Extrauterine growth retardation in premature infants in Shanghai: a multicenter retrospective review. *Eur J Pediatr* 2009;168:1055.

25. Rochow N, Landau-Crangle E, So HY, et al. Z-score differences based on cross-sectional growth charts do not reflect the growth rate of very low birth weight infants. *PloS one* 2019;14(5).

26. Sakurai M, Itabashi K, Sato Y, Hibino S, Mizuno K. Extrauterine growth restriction in preterm infants of gestational age≤ 32 weeks. *Pediatr. Int* 2008;50:70–75.

27. Simchen MJ, Beiner ME, Strauss-Liviathan N, et al. Neonatal outcome in growth-restricted versus appropriately grown preterm infants. *AM J PERINAT* 2000;17:187192.

28. Hay WW. Aggressive nutrition of the preterm infant. *Curr. Pediatr. Rep* 2013;1:229–239.

29. Andrews ET, Ashton JJ, Pearson F, Beattie RM, Johnson MJ. Early postnatal growth failure in preterm infants is not inevitable. *Arch. Dis* 2019;104:F235–41.

30. Agostoni C, Buonocore G, Carnielli VP, et al. Enteral nutrient supply for preterm infants: commentary from the European Society of Paediatric Gastroenterology, Hepatology and Nutrition Committee on Nutrition. *J. Pediatr. Gastroenterol. Nutr* 2010;50:85–91.

31. Prado EL, Dewey KG. Nutrition and brain development in early life. *Nutr. Rev* 2014;72:267–284.

Confidential: For Review Only

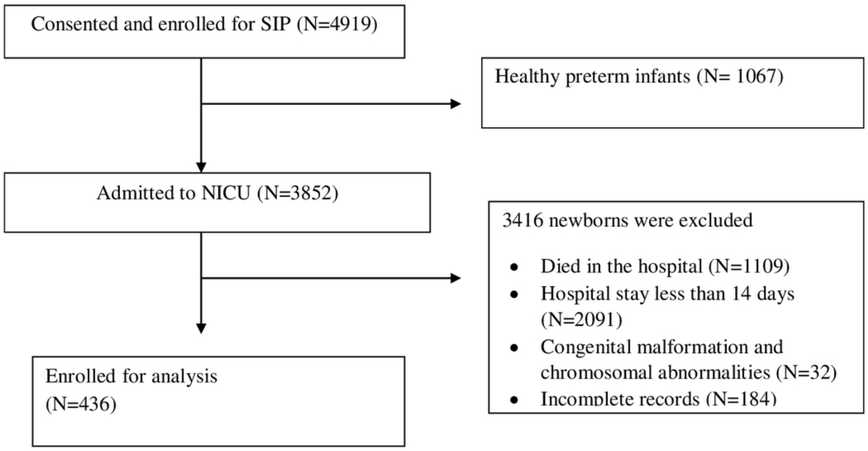

Figure 1. Flow chart of study subjects included in the analysis.

102x60mm (300 x 300 DPI)
